# Supplementary material for: Culture Degeneration Reduces Sex-Related Gene Expression, Alters Metabolite Production and Reduces Insect Pathogenic Response in Cordyceps militaris
Source: Microorganisms. 2021 Jul 22;9(8):1559. doi: 10.3390/microorganisms9081559 (PMC8400478; doi:10.3390/microorganisms9081559)
Supplement: Supplementary file 1 [file microorganisms-09-01559-s001.zip › microorganisms-1271445-supplementary.pdf]

SUPPLEMENTARY FIGURES

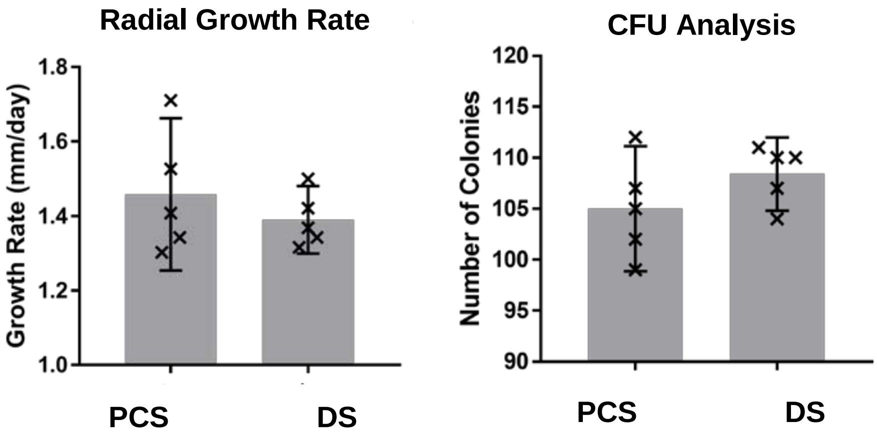

Supplementary Figure S1: Growth rates of *CM2* parental control (PCS) and degenerated (DS) strains on PDA plates. Left: radial growth rate after 20 days from subculture; right: colony forming unit analysis. Error bars show 95% confidence intervals of means, from five biological replicates.

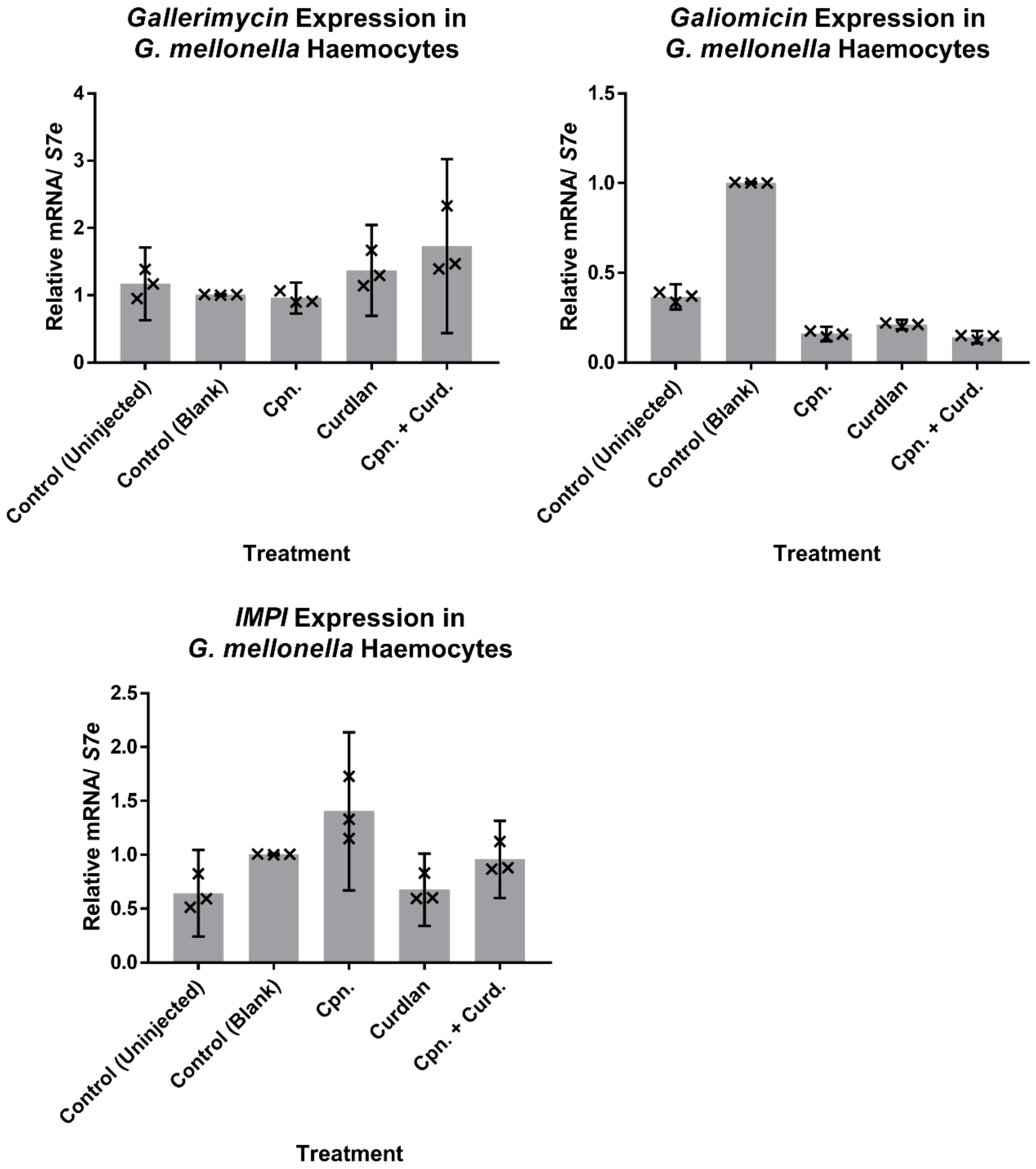

Supplementary Figure S2: ***Gallerimycin*****,** ***Galiomicin*****, and** ***IMPI*** **expression in** ***Galleria mellonella*** **haemocytes following injection by curdlan (curd.), with treatments of additional cordycepin (cpn.) and pentostatin (ptn.).** Three biological replicates, with each point an average of three technical replicates. In all cases, according to t-tests with Bonferroni corrections, there was no significant stimulation by curdlan compared to the negative controls (uninjected and blank).

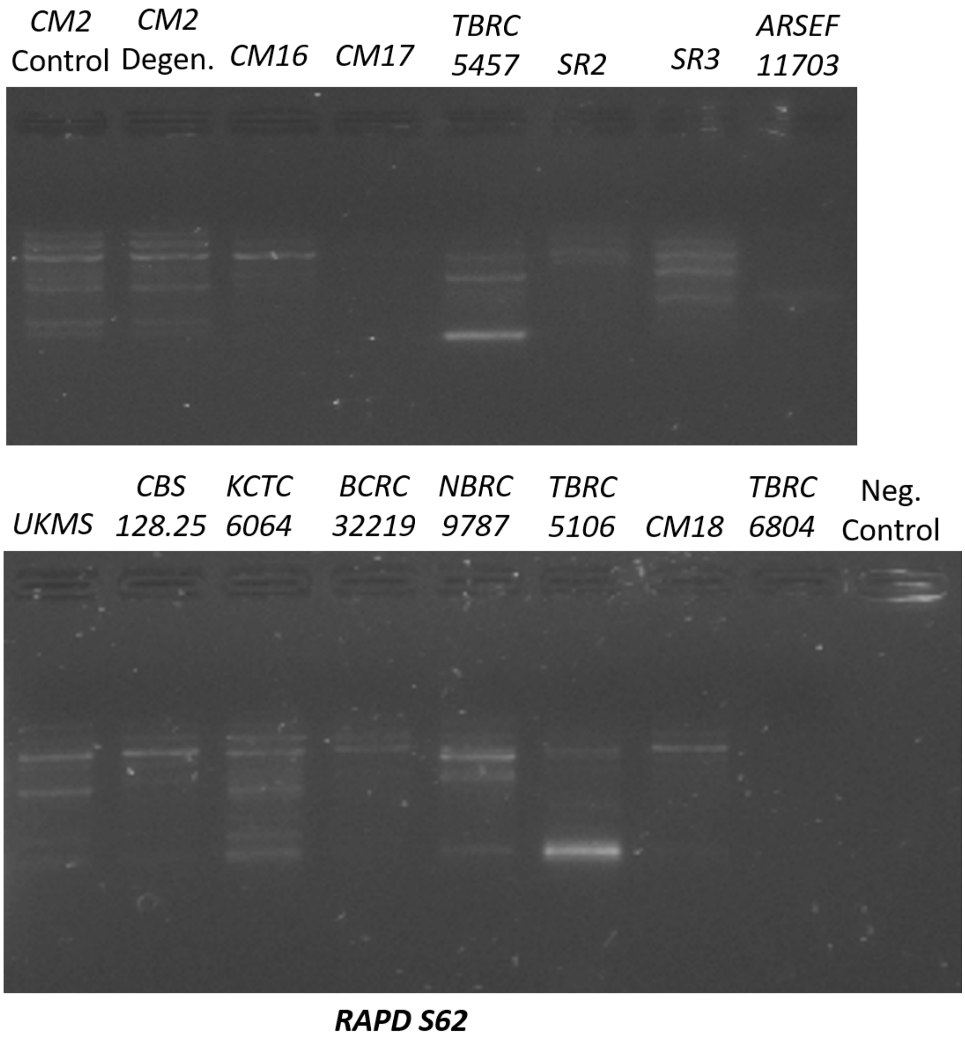

Supplementary Figure S3: RAPDs PCR screens of various *Cordyceps militaris* strains and isolates using the *RAPD S62* primers.

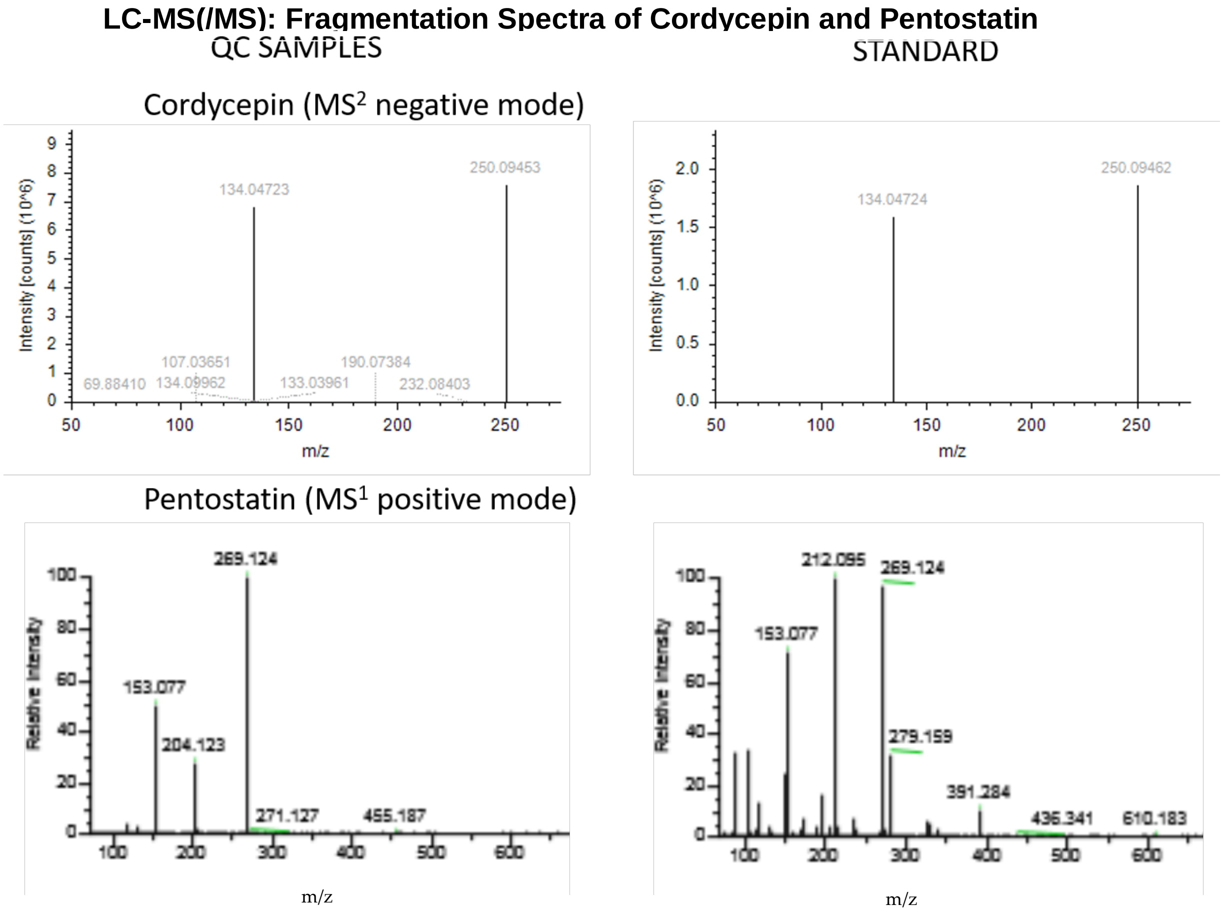

Supplementary Figure S4: Fragmentation mass spectra of cordycepin and pentostatin, using tandem MS (MS/MS) and standard MS respectively. Metabolomics Standards Initiative level 1 identification of these metabolites is confirmed by comparison of these spectra for the *C. militaris* QC samples with those of the standards used for quantification.
